# Challenges and Future Perspectives for Vaccines Targeting the Tumor Microenvironment

**DOI:** 10.3390/vaccines14070639

**Published:** 2026-07-21

**Authors:** Devin B. Lowe, Neda Feizi, Walter J. Storkus

**Affiliations:** 1 Department of Immunotherapeutics and Biotechnology, Jerry H. Hodge School of Pharmacy, Texas Tech University Health Sciences Center, Abilene, TX 79601, USA; devin.lowe@ttuhsc.edu; 2 Department of Dermatology, University of Pittsburgh, Pittsburgh, PA 15213, USA; neda.feizi@pitt.edu; 3 Departments of Immunology, Pathology and Bioengineering, University of Pittsburgh School of Medicine, Pittsburgh, PA 15213, USA; 4 UPMC Hillman Cancer Center, Pittsburgh, PA 15213, USA

Immunotherapies integrating immune checkpoint inhibitors (ICI) represent the current standard of care for many solid cancers, yet they effectively treat only a minority of patients due to intrinsic or acquired resistance mechanisms [1]. Notably, the clinical success of such immunotherapies minimally requires baseline presence of tumor-infiltrating lymphocytes (TILs) that operate within the context of a proinflammatory “hot” tumor immune microenvironment (TIME) [1,2]. To augment levels of such therapeutic immune effector cells, active vaccination approaches are warranted as priming or boosting regimens prior to the administration of ICI or for inclusion in combination interventional protocols to improve treatment overall (objective) response rates and disease outcome benefits.

Vaccines can be applied via delivery of ex vivo-generated formulations (peptide-, mRNA-, or -DNA-based) along with immunostimulatory adjuvants (i.e., conventional vaccines) or by the introduction of agents (i.e., in situ vaccines) into the TME that promote tumor immunogenic cell death and/or a proinflammatory TIME conducive to improving tumor antigen cross-presentation and T cell cross-priming/recruitment [3]. Combination vaccine approaches can further integrate (i) agents that prevent accumulation or functionality of local immunoregulatory cell types that restrict optimal anti-tumor TIL efficacy—such as Treg, myeloid-derived suppressor cells (MDSCs), or M2-like tumor-associated macrophages (TAMs)—and/or (ii) immune checkpoint inhibitors that reinvigorate the poly-functional, poly-specific anti-tumor T cell repertoire [4].

In order to optimize the tumor selectivity of therapeutic T cell responses and minimize on-target/off-cancer immune-related-adverse-event autoimmune pathologies, conventional cancer vaccines traditionally target antigens that are grossly overexpressed or evolve from gene mutation events or aberrant processing events in tumor cells (i.e., somatic neoantigens [NeoAg]) [3]. However, the tumor-conditioned microenvironment can also coordinately enforce altered epigenetic programming in its constituent (non-tumor) stromal cell populations, including cancer-associated fibroblasts, peri-vascular pericytes/mural cells, and vascular endothelial cells, leading to aberrant (over)expression of non-mutated antigens that can be recognized by T cells and B cells/antibodies that provide therapeutic benefits [5,6]. Such T cell targeting of tumor stromal cell populations is likely cogent to immunotherapy outcomes whether it occurs as a direct consequence of active specific vaccination or as an unintended but beneficial by-product of epitope spreading in the T cell repertoire initiated by treatment-associated TIME inflammation.

For this Special Issue, we welcomed articles focused on cutting-edge (ex vivo-prepared or in situ) vaccine-based approaches designed to improve the therapeutic host response against cancer when applied as single modalities or in combination protocols integrating TME-conditioning agents that mitigate immunoregulatory circuitry and/or promote enhanced recruitment and sustained fate/function of anti-tumor effector cells. We are pleased to publish 11 articles in this Special Issue, with content spanning peptide/protein-, mRNA- and DNA-based vaccines, combination immunotherapies integrating ICI and Treg antagonists, and the use of comprehensive on-treatment tumor transcriptional profiling to monitor evolving TIME changes associated with patient outcomes to inform biomarker selection and improve the design of more effective interventional immunotherapies.

## 1. Vaccines and Immunotherapies Targeting the TME

Cecil et al. [7] developed peptide-based vaccines targeting non-mutated breast cancer stem cell (CSC)-overexpressed antigens (CD105, CDH3, FOXQ1, MDM2, SOX2, and YB1) that are known to predict a higher risk of breast cancer recurrence, metastasis, and resistance to interventional therapy. Subcutaneous prime/boost vaccination with pooled peptide-based vaccines in complete/incomplete Freund’s adjuvant (CFA/IFA) induced specific Th1 CD4^+^ T cell responses in association with slowed breast cancer growth in vivo. Interestingly, despite eliciting only modest IFNγ production from primed CD4^+^ T cells, CD105-based vaccines were among the most therapeutically effective interventions in these models, a phenomenon that may relate to concomitant vaccine-induced targeting of CSC and CD105^+^ tumor-associated vascular endothelial cells (VECs) leading to suppressed tumor angiogenesis.

Using a subcutaneous DC/peptide-based immunization approach, Taylor et al. [8] similarly found that vaccines targeting non-mutated antigens overexpressed by VECs or pericytes within the TME effectively promoted tumor vascular normalization and stimulated specific polyfunctional CD8^+^ T cell responses while reducing regulatory immune cell content in association with significantly slowed established B16 melanoma growth and an extended median overall survival (OS) in vivo. The therapeutic efficacy of this vaccine formulation was improved by further inclusion of tumor intrinsic antigen (lineage-restricted or NeoAg)-derived peptides or by combination treatment with anti-PD-L1 mAb or a chemokine-modulating regimen designed to promote enhanced recruitment of vaccine-induced CD8^+^ T cells into the TME. The authors suggest that similar vaccines targeting antigens aberrantly (over)expressed by TME component adipocytes, cancer-associated fibroblasts, or mesenchymal stem cells might also represent salient interventional platforms for improving immunotherapeutic outcomes for cancer patients.

Focusing on protein-based conventional vaccines, Cheng et al. [9] report that a chimeric chaperone (Flagrp170)-TLR5 agonist-antigen complex vaccine efficiently induced activation/maturation of host Batf3^+^ dendritic cells, leading to effective antigen cross-presentation and the promotion of anti-tumor CD8^+^ T cell responses that mediate therapeutic benefits in mouse breast carcinoma and melanoma models. Therapeutic vaccination promotes a proinflammatory TIME that is far more responsive (i.e., OS is extended) to interventional anti-PD1 ICI when applied in combination immunotherapy approaches. The ability of the combined vaccine + ICI treatment approach to be curative in 60% of mice bearing B16 melanomas was particularly notable, given the notorious resistance of this model to anti-PD1 monotherapy.

Roy and Anderson [10] highlight current advances in optimizing mRNA-based cancer vaccine development/testing pipelines based on an immunologic framework integrating antigen design, delivery platforms, and immune monitoring to optimize the in vivo induction of durable, polyfunctional, poly-specific T cell responses that resist T cell exhaustion, immune suppression, and tumor evasion mechanisms. Existing strategies including mRNA vaccines + ICI or chimeric antigen receptor–T cell adoptive cell therapy have shown early clinical potential for improving patient treatment outcomes, with important issues related to future development of circular and self-amplifying RNA vaccines and logistically tenable advances in personalized vaccine-centric precision immunotherapies discussed as well.

Gazouli and colleagues [11] provide a field update on the promise and current state of personalized mRNA-based vaccine interventional approaches for melanoma patients. Thes approaches have attracted attention recently because of their ability to significantly reduce disease recurrence in patients receiving an adjuvant anti-PD-L1 monoclonal antibody (mAb) pembrolizumab + mRNA vaccine (V940; encoding a range of NeoAgs) vs. ICI alone [12]. Importantly, the authors discuss concerns for real-world application of personalized mRNA-based vaccines in lower-income countries that are limited by the requirement for state-of-the-art technologies, the high cost of vaccine production, vaccine distribution logistics, and cold-chain storage considerations.

In a commentary piece, Wang, Han, and Ling [13] suggest that the anti-tumor efficacy of mRNA-based vaccines (such as personalized mRNA-4157 or LK101) in the liver cancer setting might be improved by dietary ursodeoxycholic acid supplementation or via therapeutic delivery of bile acid-CoA:amino acid N-acyltransferase (BAAT) inhibitors to modulate bile acid metabolism. The authors posit that such combination vaccine strategies antagonize the immunosuppressive TIME driven by bile acid composition, leading to improvments in the recruitment, fate, and therapeutic action of vaccine-induced TIL, which they plan to investigate in appropriate model systems prospectively.

Raju et al. [14] developed an oral salmonella-based DNA vaccine designed to limit tumor angiogenesis via the targeting of the pro-adrenomedullin N-terminal 20 peptide (PAMP) hormone. While prophylactic vaccination in mice did not significantly prevent B16 melanoma metastasis to the lungs and overall disease progression, the authors noted the development of circulating anti-PAMP Abs and evidence of reduced angiogenesis/tumor cell proliferation and increased CD8^+^ TIL content in situ. However, vaccination also resulted in elevated Treg and Arg1^+^ M2 tumor-associated macrophage frequencies in the TME, which may represent obstacles for the optimal anti-tumor efficacy of this TME-targeted vaccine approach. Such acquired resistance mechanisms will likely necessitate further inclusion of targeted antagonists in combination vaccine approaches to enable optimal treatment-associated benefit.

In regard to studies of human papilloma virus (HPV)-related cancers, Lim et al. [15] report that HPV-16 E7 targeted DNA/electroporation-based vaccines remain immunogenic and therapeutic but with attenuated efficacy in the setting of host STAT1 deficiency (where spontaneous HPV^+^ tumor growth is accelerated and T cell exhaustion is more prevalent relative to control mice), supporting the likely protective effects of such treatment modalities in patients exhibiting partial deficits in immunocompetency. The current state of genetic (mRNA and DNA) vaccines for effective prophylaxis and therapy of HPV(16)^+^ cervical cancer was also discussed by Lien et al. [16], who identified a range of areas for future field advancements, including expanded study of vaccines’ impact on HPV-18^+^ cancers and combination vaccine protocols integrating ICIs.

## 2. Antagonizing Regulatory Immune Cells to Improve the Anti-Tumor Efficacy of Cancer Vaccines

Gwin and colleagues [17] report the results of a Phase II clinical trial of denileukin difitox (ONTAK; diptheria toxin/IL-2 fusion protein that depletes Treg) in 15 advanced treatment-refractory breast cancer patients, noting that anti-DT antibodies developed in most patients, with 40% of cases exhibiting a (≥25%) reduction in circulating levels of CD4^+^CD25^+^Foxp3^+^ Treg cells. However, stable disease was the best pathologic response achieved in four patients, with nine patients progressing to treatment and moderate immune-related adverse events (including vascular leak syndrome) observed in most patients in the trial. These findings are consistent with the results of past clinical studies failing to definitively demonstrate an objective treatment benefit for this agent when applied as a monotherapy in the advanced solid-cancer setting. These results add to an equivocal research base for this agent, which has demonstrated highly variable effects on vaccine-induced anti-tumor T cell responses in solid-cancer patients [18,19].

## 3. Longitudinal TME/TIME Gene Profiling to Improve Vaccine/Immunotherapy Clinical Outcomes

Phung, Nejo, and Okada [20] reviewed the value of comprehensive profiling of glioma tissues obtained from patients treated with immunotherapies (including a range of conventional and in situ vaccine interventions) to provide insights on how effectively drugs are delivered into the TME and how these therapies change the TIME to facilitate pathologic clinical responses to best inform the design/performance of prospective clinical trials. Within the context of glioma vaccine trials, such studies have provided instances in which treatment-induced anti-tumor T cells are coordinately detected in both peripheral blood and tumors, while in other cases, they are only found in circulation, suggesting the need for co-therapeutics to condition the TME to improve vaccine-induced T cell recruitment and the therapeutic benefit for this cohort of patients. Clinical trial designs integrating multiple longitudinal tumor biopsies generate a means of assessing TIME therapeutic dynamics and trajectories linked to preferred patient outcomes. The results of these investigations coordinately advance our understanding of immune–oncology and provide a correlative mechanistic framework for improved (and increasingly personalized) interventional protocol development and testing.
